# Coupling a Single Nitrogen-Vacancy Center in Nanodiamond to Superparamagnetic Nanoparticles

**DOI:** 10.1038/s41598-018-26633-9

**Published:** 2018-05-30

**Authors:** Nikola Sadzak, Martin Héritier, Oliver Benson

**Affiliations:** 1 Nano-Optics, Institute of Physics, Humboldt-Universität zu Berlin, Newtonstr. 15, D-12489 Berlin, Germany; 2 Department of Physics, ETH Zürich, Otto-Stern-Weg 1, 8093 Zürich, Switzerland

## Abstract

Single nitrogen-vacancy (NV) defect centers in diamond have been exploited as single photon sources and spin qubits due to their room-temperature robust quantum light emission and long electron spin coherence times. They were coupled to a manifold of structures, such as optical cavities, plasmonic waveguides, and even injected into living cells to study fundamental interactions of various nature at the nanoscale. Of particular interest are applications of NVs as quantum sensors for local nanomagnetometry. Here, we employ a nanomanipulation approach to couple a single NV center in a nanodiamond to a single few-nm superparamagnetic iron oxide nanoparticle in a controlled way. After measuring via relaxometry the magnetic particle spin-noise, we take advantage of the crystal strain *m*_*s*_ = ± 1 spin level separation to detect the superparamagnetic particle’s effect in presence of a driving AC magnetic field. Our experiments provide detailed insight in the behavior of such particles with respect to high frequency fields. The approach can be extended to the investigation of increasingly complex, but controlled nanomagnetic hybrid particle assemblies. Moreover, our results suggest that superparamagnetic nanoparticles can amplify local magnetic interactions in order to improve the sensitivity of diamond nanosensors for specific measurement scenarios.

## Introduction

Defect centers in diamond have been intensively studied due to their outstanding properties as solid-state atom-like systems. In particular, the nitrogen-vacancy (NV) center shows remarkable features as a stable single photon source emitting in the visible spectral range even at room temperature^1^ without undergoing bleaching or blinking. Moreover, the possibility of deterministically creating NV centers in diamond^2^ and its availability in nano-scaled particles paved the way towards its use as a quantum light source suitable for fundamental studies in key fields such as quantum cryptography^3^ or quantum photon interference phenomena^4,5^. Another promising research direction is the realization of hybrid structures which combine NV centers with diverse optical elements. Recent progresses in diamond fabrication techniques demonstrated waveguides and optical cavities directly in the bulk crystal^6,7^; nanodiamonds instead allow for an approach based on nanomanipulation techniques^8^, where nitrogen-vacancies are coupled to optical fibers^9^, photonic crystal cavities^10^ or plasmonic elements^11,12^, and as well used as scanning probes for characterizing nanoscale processes in light-matter interaction^13^. The possibility of initialization, manipulation and readout of the defects’ electron spin allows its use as a spin qubit^14^ or nanomagnetometer^15^. Concerning the latter, several applications have been so far demonstrated, including the detection of single electron spins within the diamond lattice^16^, single surface molecules^17^ or single magnetic nanoparticles^18^. Moreover, NV centers have been used to measure the mesoscopic spin dynamics and spin texturing of ferrimagnetic^19^ and anti-ferromagnetic thin films^20^. In this work, we use a bottom-up approach to combine a single nitrogen-vacancy center in a nanodiamond with a single superparamagnetic iron oxide nanoparticle (SPION) as a first step towards creating hybrid nanodiamond/magnetic particle structures. Superparamagnetic nanoparticles are single-domain magnetic systems whose properties have been exploited in a broad range of technological applications^21^, from magnetic information storage^22^ to ferrofluids^23^ or nanoscale drug-delivery systems^24^ and magneto-assisted hyperthermia cancer treatments^25^. While nanoscale magnetic materials have been already studied for a long time^21^, single SPIONs were recently detected via bulk diamond NV center magnetometry using a combination of relaxometry and Hahn-echo measurements^18^ while small aggregates of maghemite superparamagnetic nanoparticles have been detected via relaxometry with a scanning ensemble of NVs^26^. We prove that such detection can be successfully performed with a nanodiamond embedded defect center by using a platform which combines confocal microscopy with electron spin manipulation and atomic force microscopy (AFM) manipulation. Moreover, by taking advantage of the crystal strain, we are capable to perform our measurements also without external DC magnetic fields generally required to address individual electron spin levels^27^. Finally, we observe the AC magnetic field response of a SPION and its effect on the NV center coherent electron spin transitions, and discuss briefly which applications can be foreseen in the quantum sensing field.

## Results and Discussion

**Figure 1:**
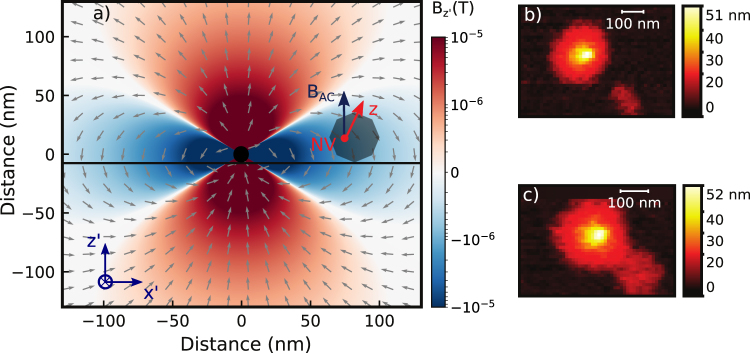
(**a**) Simulation showing the spatial distribution of the magnetic field generated by a 11 nm wide spherical single magnetite nanoparticle (black dot in the center) driven by a 12.0 G field oriented along the *z*′ direction (orthogonal to the sample surface). The *xy*-component of the particle response (or AC magnetization) has an intensity of several mG even at a 100 nm separation, but decreases to less than 0.5 mG for distances higher than 250 nm. (**b**) AFM scan of a $\simeq$50 nm nanodiamond and a 11 nm SPION with a separation >300 nm. (**c**) AFM scan after the SPION nanomanipulation showing a separation <100 nm. The xyz reference system is defined by the NV center, with its quantization axis being z, while the x’y’ reference system is parallel to the waveguide surface and defined by the Helmholtz coils. The MW field is parallel to z’.

**Figure 2:**
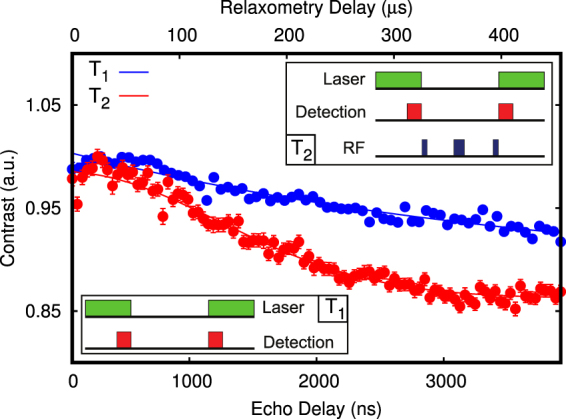
Relaxometry and Hahn-echo measurements taken on a bare single NV center in nanodiamond. A longitudinal decay time of ${T}_{1}=474\pm 136$*μ*s and a Hahn-echo transverse decay time of ${T}_{2}\mathrm{=1.74}\pm 0.06$ *μ*s was observed. The pulsed sequences are shown as well: in the *T*_1_ measurement (blue dots), two laser pulses are used to polarize the NV electron spin in *m*_*s*_ = 0 and detect its remaining polarization after a certain free evolution time *τ*. In the Hahn-echo *T*_2_ measurement (red dots), a polarizing laser pulse is followed by three resonant microwave pulses (a *π*/2 pulse, a *π* pulse and finally a *π*/2). During this sequence the NV electron spin accumulates a phase proportional to the strength of oscillating magnetic fields acting along the defect axis. A last laser pulse is used to detect the final magnetometer state at the end of the measurement.

**Figure 3:**
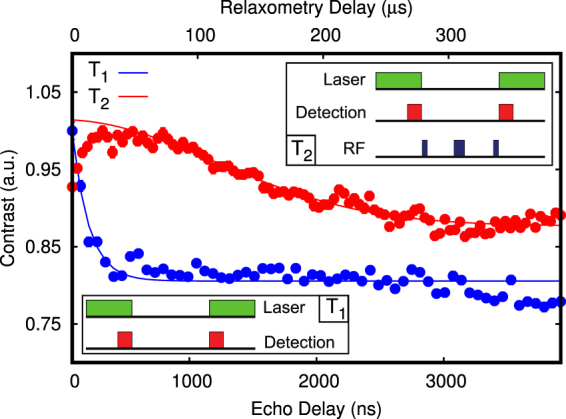
Relaxometry of the *m*_*s*_ = 0 spin state and Hahn-echo measurement for the NV center with a proximal superparamagnetic nanoparticle (red dots and line) located at <80 nm: in absence of driving magnetic fields, the high frequency particle magnetic noise induces a strong decay of the *T*_1_ coherence time from 474 ± 136 *μ*s to 13 ± 2 *μ*s, while the Hahn-echo shows a negligible change (to 1.69 ± 0.07 *μ*s) due to the intrinsic high dephasing rate of the NV in the nanodiamond.

We start our experiment with a prepared microwave coplanar waveguide (MW-CPW) patterned glass support (see methods and supplementary) where a selected nanodiamond containing a single NV center was positioned by AFM manipulation in proximity (slightly more than 700 nm) to a small cluster of previously spin-coated SPIONs (less than 8 nanoparticles). The residual magnetic field from the SPION cluster at the location of the NV center is in the range below 2 mG (see Fig. 1). This is a negligible contribution to the local magnetic environment of the NV center. For this reason we denote the NV as ‘bare’ NV center in this situation. We now proceed in characterizing the magnetic environment of this bare defect. By acquiring a Hahn-echo decay of the electron spin, we obtain the T_2_ dephasing time; the same measurement provides as well sensitivity to low frequency AC magnetic fields parallel to the NV center quantization axis^28^. With the bare nanodiamond, the T_2_ time is 1.74 ± 0.06 *μ*s (see Fig. 2). This is compatible with our specific NV physical environment, that is a non-isotopically pure type Ib nanodiamond: in this case, the sub-microsecond inhomogeneous dephasing times are due to the high concentration of nitrogen substitutional impurities as well due to surface spin fluctuations^29,30^. Fast varying magnetic fields with a frequency comparable to the NV center Larmor frequency and oriented orthogonally to the NV center quantization axis can be detected via electron spin relaxometry^31,32^. For our nanodiamond, T_1_ electron spin relaxometry of the *m*_*s*_ = 0 state (Fig. 2) gives a longitudinal decoherence time of 474 ± 136 *μ*s, compatible with previous studies on nanodiamonds indicating surface paramagnetic impurities being a major source of magnetic noise^33^. Furthermore, by using the 2D static magnetic field provided by the Helmholtz coils we estimate the NV center axis to have a deviation angle from the x’ coil axis corresponding to 72 ± 1 deg. Finally, coherent electron spin Rabi oscillations were driven with a constant microwave power resulting in a ${T}_{2}^{\ast }$ time of 550 ± 50 ns and a 7.33 ± 0.05 MHz frequency. As the MW field is perpendicular to the coplanar waveguide surface, we estimate its angle with respect to the NV center quantization axis to be *ϕ* = 18 ± 1 deg. The MW driving field magnitude can be then extracted from the Rabi oscillation frequency since (with a zero detuning) ${\omega }_{R}={\gamma }_{NV}{B}_{AC,xy}$ and ${B}_{AC,xy}=\frac{1}{\sqrt{2}}{B}_{AC}sin(\varphi )$, with ${\gamma }_{NV}\mathrm{=2}\pi \times 2.8$ MHz/G being the gyromagnetic ratio of the NV center and *B*_*AC*_ the MW field. From the obtained 7.33 ± 0.05 MHz Rabi frequency *ω*_*R*_, we estimate *B*_*AC*_ to be 12.0 ± 0.1 G. With these measurements all the properties of the bare NV center are fully characterized. Now we proceed with measuring the effect of coupling to a SPION in a precisely controlled way. In order to achieve this we separate an individual SPION of diameter 11 ± 1 nm from the cluster of magnetic nanoparticles ~700 nm away from the NV center on the same CPM support by AFM manipulation^34^. The SPION is then moved to a distance of 50 to 100 nm from the nanodiamond (Fig. 1b and c). We first proceed to perform a relaxometry measurement. Here, for a separation lower than 100 nm, a substantial reduction of the T_1_ time is observed down to 13 ± 2 *μ*s, that is an order of magnitude lower than the bare NV center in the nanodiamond (Fig. 3).

**Figure 4:**
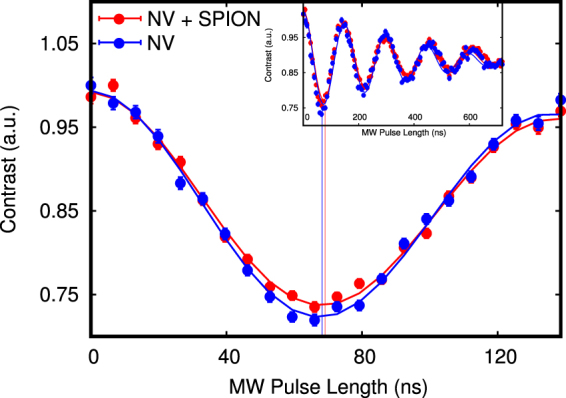
Precise measurement of Rabi oscillations for the 2.855 GHz transition driven by a constant external AC magnetic field with the bare NV center (blue dots) and the NV center interacting with a proximal superparamagnetic nanoparticle (red dots), with the solid lines representing the data fits (via an exponentially decaying cosine function). The frequency difference is on the scale of ${\rm{\Delta }}{\omega }_{R}$ = 90 ± 70 KHz, going from 7.33 ± 0.05 MHz with the bare NV center to 7.24 ± 0.05 MHz for a mutual NV-SPION separation of 64 ± 3 nm and a NV axis - SPION angle of 72 ± 1 deg. The full envelope of the Rabi oscillations is shown in the upper frame.

The T_2_ dephasing time, measured by means of Hahn-echo (Fig. 3), is negligibly affected, as it mainly arises from the coupling to the substitutional nitrogen spin bath, whose interaction strength is more than an order of magnitude higher than the particles magnetic noise strenght. As a second experiment we apply an external oscillating RF field and measure Rabi oscillations. We find that by keeping the MW power constant, Rabi oscillations are frequency shifted to 7.24 ± 0.05 MHz from the initial 7.33 ± 0.05 MHz, so ${\rm{\Delta }}{\omega }_{R}$ = 90 ± 70 KHz slower than reported in the reference measurement (see Fig. 4). In the following we proceed to explain our observations, which are related to the NV center being in close proximity to the SPION particle (since any other experimental parameter is kept constant) in the absence of driving fields (relaxometry) and with an external oscillating RF field (Rabi oscillations). The iron oxide nanoparticle is an FCC-ordered ferrimagnetic ensemble of iron and oxygen atoms, where the net magnetization per lattice unit is given by 8 Iron $F{e}^{2+}$ atoms occupying octahedral sites^35,36^. Due to the magnetocrystalline anisotropy arising from spin-spin coupling, the iron magnetic moments tend to be preferentially aligned (above the Vervey transition temperature) along the [111] crystal axis and its eight geometrical equivalents for a cubic lattice^37,38^. Moreover, below a certain material dependent size, these magnetic moments are arranged in a single domain^39,40^ and behave collectively as a single magnetic dipole. At a given instant, the total magnetic field B generated by the latter can be obtained by integrating the magnetic moments of each crystal unit cell over the particle volume:

1 $${\bf{B}}({\bf{r}})=\frac{{\mu }_{0}}{4\pi }[-\frac{{\bf{m}}}{{{\bf{r}}}^{{\bf{3}}}}+\frac{{\bf{3}}({\bf{m}}\cdot {\bf{r}}){\bf{r}}}{{{\bf{r}}}^{{\bf{5}}}}]$$

where **m** is the total SPION magnetic moment, calculated by summing the magnetization of each unit cell (that is 32 *μ*_*b*_)^35^ over the nanoparticle volume, given a unit cell length of 0.839 nm. In a very weak $(mB\ll KV)$ or absent magnetic field, the particle magnetization direction fluctuates in space on a time scale *τ*_*N*_ defined by the Neel relaxation time^41^ giving origin to the phenomena of superparamagnetism:

2 $${\tau }_{N}={\tau }_{0}\exp (\frac{KV}{{k}_{b}T})$$

where *τ*_0_ is a material-dependent parameter and has generally values between 10^−9^ and 10^−13^ seconds^42,43^, K is the magnetocrystalline anisotropy constant assumed to be 14 KJ/m^3^ ^44^ and V is the particle volume. According to Brown^45^, the magnetization vector motion follows a random stochastic process. By assuming the SPION magnetization dynamics to be on a faster timescale than the NV spin relaxation and to be governed uniquely by a Neel-Arrhenius behavior, we can describe the magnetization autocorrelation function as an exponentially decaying function having a characteristic time ${\tau }_{c}\approx {\tau }_{N}$. This leads us to express the modified T_1_ relaxation rate^46,47^ as:

3 $$\frac{1}{{T}_{1}}=\frac{1}{{T}_{\mathrm{1,}int}}+3{\gamma }_{NV}^{2}\langle {B}_{xy}^{2}\rangle \frac{{\tau }_{N}}{1+{\omega }_{0}^{2}{\tau }_{N}^{2}}$$

where *T*_1_ is the total relaxation rate, ${T}_{\mathrm{1,}int}$ is the intrinsic relaxation rate of the bare NV in the nanodiamond, ${\gamma }_{NV}$ is the NV gyromagnetic ratio of the free electron expressed here as $2\pi \times 2.8MHz/G$, ${\omega }_{0}$ the resonant ${m}_{s}=0\to {m}_{s}=-\,1$ NV transition frequency of $2\pi \times 2.855MHz$, and $\langle {B}_{xy}^{2}\rangle$ is the variance of the SPIONs magnetic field projection on the NV center *xy* plane for all the possible angles of the SPION magnetization (see supplementary material). By using the *T*_1_ times obtained from the experiment and shown in Fig. 3, and calculating a ${\tau }_{N}\mathrm{=5.5}\times {10}^{-11}$ s from a ${\tau }_{0}\mathrm{=5}\times {10}^{-12}$ s, we obtain a $\langle {B}_{xy}^{2}\rangle$ of 2.9 ± 0.5 *G*^2^, which corresponds to an NV-SPION separation of 64 ± 3 nm, given a particle radius of 5.5 nm (see further details in the supplementary). This is indeed nicely compatible to the configuration realized in our experiment as depicted in Fig. 1b and c. While the relaxometry measurement served to probe the noise generated by the zero-averaging SPION fluctuating magnetization in absence of an external field, the measurement in the presence of a driving AC field is affected by the net SPION magnetization. Here the crystal strain, as well as a small residual magnetic field, is lifting the ${m}_{s}=\pm 1$ degeneracy establishing a two-level system interacting with the AC field. The sensitivity of the observed Rabi oscillations is limited by ${T}_{2}^{\ast }$ and maximized when ${\omega }_{AC}\simeq {\omega }_{0}$.

However, only the magnetization component transversal to the NV center axis couples to its spin. Describing analytically the magnetization vector motion in space is a very complex task. Instead, we calculate its average values which are observed during a driving microwave pulse. In this case, the magnetization behavior results in a combination of fluctuations around the magnetocrystalline anisotropy axis due to thermal relaxation, and alignment with respect to the driving field due to the latter interacting with the particle moment. At zero frequency (DC) the equilibrium magnetization is described by the Langevin equation^48^:

4 $${\langle m\rangle }_{B}=m[\coth (\frac{mB}{{k}_{b}T})-{(\frac{mB}{{k}_{b}T})}^{-1}]=mL(\frac{mB}{{k}_{b}T})$$

where *m* is the particle total magnetic moment, *T* the temperature and *B* the external field. At non-zero (AC) frequencies instead, the Casimir-DuPre model^49^ can be used to derive a complex susceptibility via a Debye-like approach, where the particle’s in-phase linear response leads to a magnetization amplitude defined as:

5 $${\langle m\rangle }_{{B}_{AC}}=m\frac{L^{\prime} (m{B}_{AC}/{k}_{b}T)}{1+{\omega }_{0}^{2}{\tau }_{N}^{2}}{B}_{AC}$$

with *ω*_0_ being the field frequency (resonant to the NV center spin transition) as previously defined, L’ is the derivative of the Langevin function with respect to the magnetic field and *τ*_*N*_ the Neel relaxation time. Combining the previous equation with the equations (1) and (4) allows us to simulate the AC magnetization effect on the NV center spin. From the experimental results as from Fig. 4 we were able to measure ${\rm{\Delta }}{\omega }_{R}\mathrm{=90}\pm 70$ KHz, corresponding to a ${B}_{AC,xy}$ field intensity decrease of 45 ± 35 mG. This agrees with the simulation of a 11 nm wide SPION subject to a 12.0 G strong AC field, that shows an effective magnetization of $\langle m\rangle \approx 1.3\times {10}^{3}{\mu }_{B}$ and at a distance of 64 nm generates a *z*′-oriented in-phase magnetic field in the range of 23 mG (see supplementary material). In the experiment care had to be taken to consider fluctuations of the microwave power and stability of the spin transition resonance frequency. To check the latter, we sampled it over the course of the experiment and find it stable in the range of [2854.5, 2854.9] MHz. This allowed us to exclude detuning from the resonance line as a cause of the observed Rabi frequency variations.

## Conclusion

We succeeded in coupling deterministically a single superparamagnetic iron oxide nanoparticle to a single nitrogen-vacancy center in a nanodiamond in a controlled way. By choosing a convenient configuration of the applied AC and DC magnetic fields, we were able to perform relaxometry and Hahn-echo measurements to probe the magnetic noise generated by the SPION. Moreover, by switching to an AC-only type of magnetic perturbation, we are able to observe a net average superparamagnetic nanoparticle magnetization at the NV center Larmor frequency, inducing a decrease in the NV electron spin Rabi oscillation rate. Our work is set toward getting a deeper insight into complex hybrid NV-SPION nanostructures. In such nanostructures magnetic nanoparticles could be employed as local amplifiers of magnetic interactions to improve diamond nanosensors where low coherence times are representing a limiting factor and cannot be extended to enhance the measurement sensitivity.

## Methods

**Figure 5:**
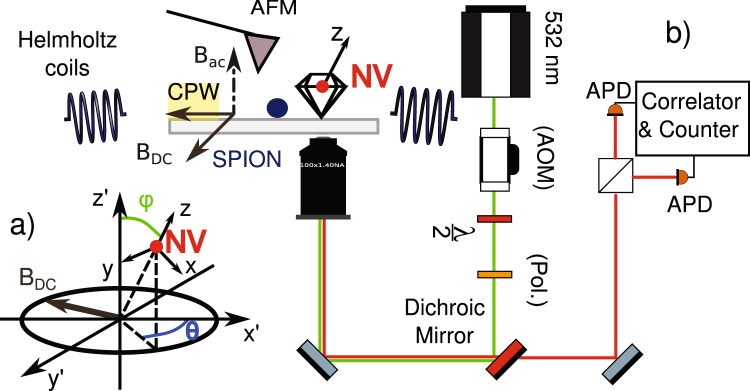
Setup schematic: a 532 nm Laser, pulsed with an acousto-optic modulator (AOM), is used to excite the NV center via a high numerical aperture (NA = 1.40) objective. The nanodiamond is positioned by means of AFM assisted pick-and-place on a glass support photolithographically patterned with a gold impedance matched coplanar waveguide (CPW, depicted as a yellow rectangle on the grey cover slide) where a diluted suspension of iron oxide superparamagnetic nanoparticles was precedently spin-coated. The coplanar waveguide generates the radio frequencies used to drive the NV center spin transitions. Two pairs of Helmholtz coils provide a static magnetic field up to 40 G per axis along the coordinates defined as *x’y’* and parallel to the support, while the CPW delivers an AC magnetic field aligned with *z’* and orthogonal to the support. The NV center defines the coordinate system *xyz* as depicted in a). The fluorescence is collected by the high NA objective and sent to an Hanbury-Brown-Twiss section where two single photon Avalanche Photodiodes (APDs) perform the intensity correlation measurement. The AFM can be used to manipulate a single iron oxide nanoparticles (SPION - depicted as a blue circle) and couple it in a controlled way to the NV center in the nanodiamond.

**Figure 6:**
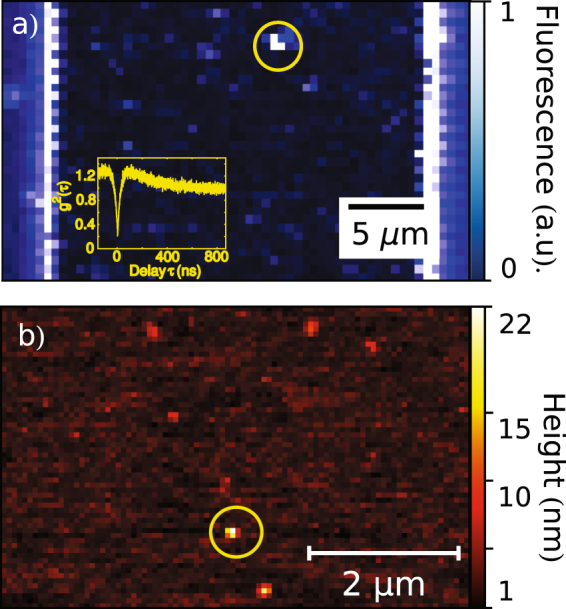
(**a**) Typical confocal scan of a coplanar waveguide where its reflective metal edges are visible as vertical bright lines and the AFM-placed nitrogen-vacancy center appears as a luminescent spot nearby the waveguide center (yellow circle). Superparamagnetic iron oxide nanoparticles, which are as well present on the sample, do not fluoresce and hence do not show up in the image. (**b**) Surface AFM raster scan of a similarly prepared CPW showing different features which correspond to single and clusters of superparamagnetic nanoparticles. The nanodiamond (yellow circle) can be identified by superimposing the topography pictures of a sample with its confocal fluorescence scans.

The experiment (see Fig. 5) was performed on a support consisting of a silicon dioxide 0.15 mm thick cover slide photolithographically patterned with a gold impedance matched microwave coplanar waveguide (MW-CPW). By using the selected geometry, the AC magnetic field has an orthogonal direction to the support surface^50^. The support was cleaned with Piranha solution (3:1 sulphuric acid/30% hydrogen peroxide) followed by sonication in a 1% Hellmanex III cleaning solution and sonication in distilled water, in order to remove any organic residuals or contaminating particles. An atomic force microscope (AFM - JPK Instruments NanoWizard) mounting an Arrow-FM silicon tip was used to check the surface after such procedure. Type Ib diamond nanoparticles (Quantum Particle 25 from Microdiamant) with an average diameter of 27 nm were used together with (10 ± 1) nm sized superparamagnetic iron oxide nanoparticles (Sigma-Aldrich). The iron oxide nanoparticle suspension was diluted with toluene and spin-coated on the support surface with an appropriate concentration for obtaining a density of few particles per 10 *μm*^2^. Further AFM images taken on different surface areas served to assess the particles distribution (see *i.e*. Fig. 6).

The CPW cover slide was mounted on a PCB featuring four calibrated Helmholtz coils capable of producing a 2D static magnetic field up to 40 Gauss per axis, and assembled on the top of a confocal microscope featuring an Olympus UPLANSAPO 100× oil immersion objective (numerical aperture NA = 1.40). A second CPW support was prepared as previously described, but having only QP25 diamond nanoparticles spin-coated on its surface instead of iron oxide nanoparticles. By using a pick-and-place procedure as described in the literature^8^, a single pre-characterized nanodiamond containing a single NV-center was then deterministically transferred from this support. For this purpose the AFM was equipped with platinum-coated NSC35/Pt tips (Nano and More) and the NV center fluorescence intensity and second order autocorrelation functions were used to monitor the pick-and-place procedure success. On the primary CPW cover slide, the transferred nanodiamond can be effectively distinguished from the iron-oxide nanoparticles, by combining surface raster-scans, fluorescence intensity maps and photon statistics measurements as shown in Fig. 6. Now, all the particles can be physically manipulated via the provided AFM featuring FM-Arrow (Nano and More) silicon cantilevers. Furthermore, the NV center spin manipulation can be performed via microwave pulses delivered by the CPW. The 2D-magnetic field generated by the two Helmholtz coil pairs allows the determination of the NV center axis orientation with respect to the coils’ plane (see supplementary material), and indirectly with respect to the driving AC microwave field (see Fig. 5). It is hence possible to assemble arbitrary 2-dimensional hybrid structures made of nanodiamonds and magnetic nanoparticles, where the nitrogen-vacancy center acts as a local magnetic-field probe capable of operating in DC and AC mode. Such approach may be used either to study the magnetic properties of complex nanoparticle architectures or to utilize the SPION interaction with the NV center to boost the sensitivity in AC magnetic measurements.

### Data availability Statement

All data generated or analysed during this study are included in this published article (and its Supplementary Information files).

## Electronic supplementary material

Supplementary Information
