# Supplementary material for: Coupling a Single Nitrogen-Vacancy Center in Nanodiamond to Superparamagnetic Nanoparticles
Source: Sci Rep. 2018 May 30;8:8430. doi: 10.1038/s41598-018-26633-9 (PMC5976675; doi:10.1038/s41598-018-26633-9)
Supplement: Supplementary file 1 — Supplementary Information [file 41598_2018_26633_MOESM1_ESM.pdf]

Supplementary Information: Coupling a Single Nitrogen-Vacancy Center in Nanodiamond to Superparamagnetic Nanoparticles

Nikola Sadzak[1,*], Martin Héritier[2], and Oliver Benson[1]

[1]Nano-Optics, Institute of Physics, Humboldt-Universität zu Berlin, Newtonstr. 15, D-12489 Berlin, Germany
[2]Department of Physics, ETH Zürich, Otto-Stern-Weg 1, 8093 Zürich, Switzerland
[*]sadzak@physik.hu-berlin.de

Experimental Setup

The confocal setup as in the main text Figure 5 has been integrated with a section that generates the microwave and radio frequency pulses necessary to perform the active manipulation of the nitrogen-vacancy center spin. Here, we used a Hameg 8135 signal generator to provide continuous-wave radio frequencies that were pulsed via a ZASW-2-50DR+ Minicircuits fast switch (5 ns of typical rise/fall time), then amplified by a high-power ZHL-16W-43+ 16-Watt Minicircuits amplifier and finally sent to the impedance matched coplanar waveguide, which is terminated by a Rhode & Schwarz spectrum analyzer. The fluorescence intensity detection was done by two Perkin Elmer Single Photon Avalanche Photodiodes (SP-APD) in a Hambury-Brown-Twiss configuration, that allowed us to record second-order fluorescence autocorrelation functions via a PicoHarp 300 correlator (PicoQuant). The pulses generated by the SP-APDs were sent to a National Instruments Data Acquisition Card (NI-DAQ 6225) which was gated via bit-pattern generator. A 166 MHz Deditec Bit-Pattern generator was used to synchronize all the devices and generate the pre-programmed TTL pulse patterns used to control the NI-DAQ card, as well as the radio frequency switching unit.

Sample Preparation

Our supports were prepared on silicon dioxide cover slides, where a coplanar waveguide was produced on one side via photolithography and subsequent sputtering of chromium and gold (Cr/Au/Cr). These supports were then cleaned in Pirahna solution for 20 minutes (3:1 sulphuric acid / 30% hydrogen peroxide) and then sonicated for 30 minutes in a 1% Hellmanex III solution and as well for 15 minutes in distilled water. In order to prepare the nanodiamond sample, we have taken a Microdiamant QP25 nanodiamond suspension (average particle diameter of 27 nm, density of 3.9 mg/ml) and diluted it with distilled water in the ratio of 1:10. Then, after sonication for 30 minutes, we have spin coated 20 μl of the diluted diamond suspension on a coplanar waveguide at 3000 rpm for 10 seconds and a 5 second acceleration; with this approach, we were able to identify single nanodiamonds via atomic force microscopy, and single nitrogen-vacancy centers via confocal microscopy. By combining the two pictures and acquiring fluorescence autocorrelation functions, we were able to single out diamond nanocrystals containing single NVs, that were then individually transferred via AFM pick-and-place to the superparamagnetic iron oxide nanoparticles (SPION) support. Concerning the latter, we prepared it separately by taking a Sigma-Aldrich SPION suspension (particles between 9 and 11 nm of diameter) having a density of 5 mg/ml and diluted it with toluene on a 1:100 ratio. After sonicating the suspension for 30 minutes, we have taken a clean CPW cover slide and spin-coated on it a droplet of diluted SPION suspension (approx 10 to 20 μl) at 3000 rpm for 15 seconds (with the rotation already in progress). By proceeding in this way, the support would show through AFM imaging a SPION density of few particles per 10 μm^2 to few particles per μm^2, although the surface would present areas with slightly higher and lower densities of particles and particle clusters.

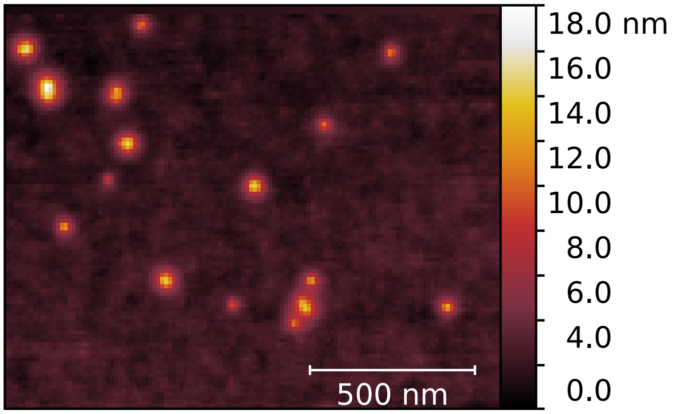

Figure S1. AFM scan of a coplanar waveguide showing a denser distribution of superparamagnetic nanoparticles. In this specific image, 7 particles show an height up to 12 nm, while one is about 18 nm and hence more likely to be a cluster of SPIONs.

NV Center Orientation

The nitrogen-vacancy center may be aligned along 4 principal diamond crystal axes[1], which are equivalent to the [111] lattice orientation and define the defect's main quantization axis. While CVD monocrystalline bulk diamond, by being generally grown along [100] or [111] planes, provides already a reference for the quantization axis direction of the NVs, nanodiamonds have their lattices randomly arranged in space and the defects embedded within them are thus randomly oriented too. In order to take advantage of the NV center as a vector magnetometer, it is useful to know its quantization axis orientation with respect to the physical objects and forces involved in the measurements. It is possible to show that a pair of Helmholtz coils, producing a two-dimensional magnetic field, is sufficient to perform such an estimation. The treatment may start with expressing the NV center Hamiltonian as:

$$H = \mathscr{D}_0 S_z^2 + E\left(S_x^2 - S_y^2\right) + \gamma_{NV} BS \quad (1)$$

Where D_0 is the zero-field splitting of 2.87 GHz, E is the transversal (strain-related) splitting, γ_{NV} the NV center gyromagnetic ratio of 2.8 MHz/G, B the external magnetic field and S the Pauli matrices representing the electron spin. In the spin-1 basis we can write the previous equation as:

$$H = \begin{pmatrix} D_0 + \gamma_{NV} B_z & \gamma_{NV} \frac{B_x - iB_y}{\sqrt{2}} & E \\ \gamma_{NV} \frac{B_x + iB_y}{\sqrt{2}} & 0 & \gamma_{NV} \frac{B_x - iB_y}{\sqrt{2}} \\ E & \gamma_{NV} \frac{B_x + iB_y}{\sqrt{2}} & D_0 - \gamma_{NV} B_z \end{pmatrix} \quad (2)$$

By assuming the transversal component of the magnetic field to be weak in comparison to the zero-field splitting, so $\gamma_{NV}\sqrt{B_x^2 + B_y^2} = \gamma_{NV} B_\perp << D_0$, we can rewrite the Hamiltonian as:

$$H = \begin{pmatrix} D_0 + \gamma_{NV} B_z & 0 & E \\ 0 & 0 & 0 \\ E & 0 & D_0 - \gamma_{NV} B_z \end{pmatrix} \quad (3)$$

having the eigenenergies:

$$\nu_{\pm 1} = D_0 \pm \sqrt{\gamma_{NV}^2 B_z^2 + E^2} \quad (4)$$

Now, we can define a system of reference for the NV center (see Figure S2), where the z-axis is parallel to the NV quantization axis, and a system of reference for the two pairs of Helmholtz coils, where x' and y' are the two coil axes corresponding to the two orthogonal magnetic field vectors $B_{x'}$ and $B_{y'}$.

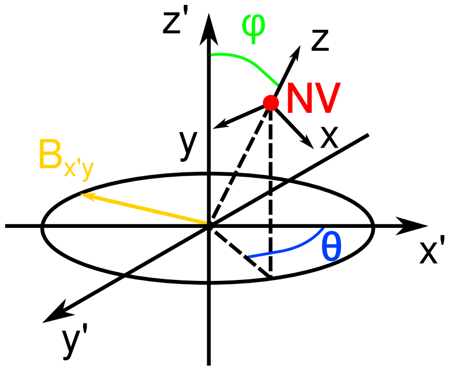

Figure S2. Sketch of the two reference systems used in the orientation measurement, where the NV center has its quantization axis defined along z, and the Helmholtz coils are generating DC magnetic fields respectively along x' and y', or if combined in the $x'y'$ plane.

These two reference systems are related through rotation operators around the polar angle ϕ and the azimuthal angle θ:

$$\begin{pmatrix} B_x \\ B_y \\ B_z \end{pmatrix} = \begin{pmatrix} cos(\phi) & 0 & sin(\phi) \\ 0 & 1 & 0 \\ -sin(\phi) & 0 & cos(\phi) \end{pmatrix} \begin{pmatrix} cos(\theta) & -sin(\theta) & 0 \\ sin(\theta) & cos(\theta) & 0 \\ 0 & 0 & 1 \end{pmatrix} \begin{pmatrix} B_{x'} \\ B_{y'} \\ B_{z'} \end{pmatrix} \tag{5}$$

By working in the low-field regime, we can assume that the $\nu_{\pm 1}$ splitting is solely related to B_z; moreover, as the pairs of Helmholtz coils generate by definition of the reference systems only magnetic fields parallel to x' and y', we can set $B_{z'} = 0$. Hence, the eigenenergies of the Hamiltonian (1) can be written as:

$$\nu_{\pm 1} = D_0 \pm \sqrt{\gamma_{NV}^2 \left(B_{y'} sin(\theta) sin(\phi) - B_{x'} cos(\theta) sin(\phi) \right) + E^2} \tag{6}$$

In the particular case where $\theta \approx \pi/2$ (that means, one of the Helmholtz coil pairs generates a field orthogonal to the NV quantization axis) the expression is further simplified to:

$$\nu_{\pm 1} = D_0 \pm \sqrt{\gamma_{NV}^2 B_{y'} sin(\phi) + E^2} \tag{7}$$

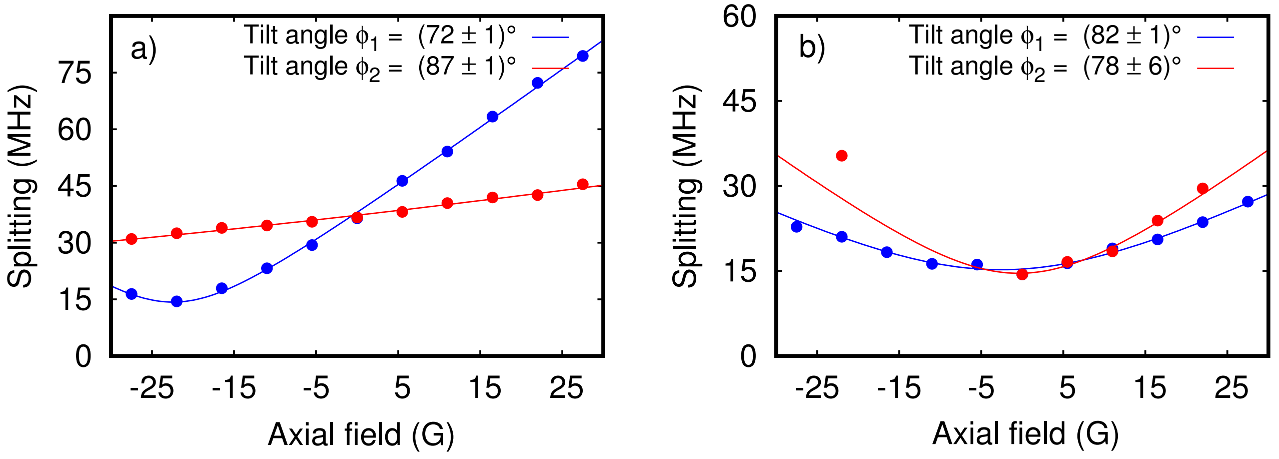

Figure S3. Plots showing the NV center energy splittings between the ground state spin sublevels for two different nanodiamonds as a function of the DC magnetic field generated along x' (blue dots) and y' (red dots) with the tilt angles obtained through the fit functions derived from the equations (6) and (7) (blue and red lines respectively for the x' and y' oriented coils). The angles ϕ_1 and ϕ_2 are defining the orientation between the DC field axes (and the sample) and the NV center axis. It can be observed that in a) the NV center axis lies almost parallel to y' and moreover a static residual field of < 7 G is present, while in b) this field is zero.

This condition is verifiable by sweeping the $B_{x'}$ field and noticing that the effect on the detuning is negligible as shown in Figure (S3a): from the same measurement we deduce that while approaching orthogonality to $B_{x'}$, the NV center quantization axis has an angle of 72 ± 1 deg with respect to $B_{y'}$ and hence of 18 ± 1 deg with respect to the z' axis. Figure S3b shows instead the case where $\gamma_{NV} B_{y'} \neq 0$ and $\gamma_{NV} B_{x'} \neq 0$. In this case, the assumption of orthogonality does not hold anymore.

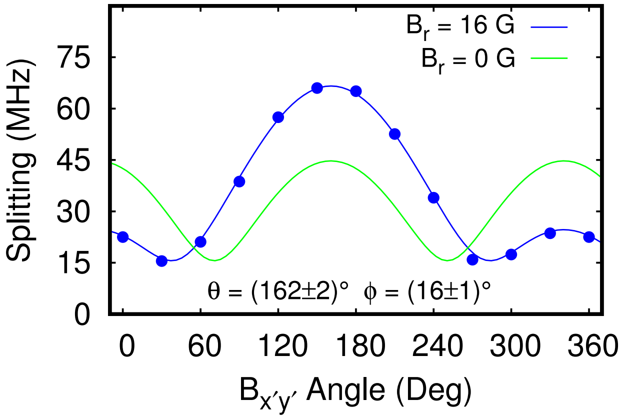

Figure S4. Energy separation (blue dots) between the NV center spin sublevels for a DC magnetic field generated on a circumference in the $x'y'$ plane with a constant magnitude. Fitting the data (blue line) allows us to retrieve the angles of the NV center axis with respect to the experimental system of reference as defined in Figure S2. The green line shows the fit result in absence of a third residual magnetic field acting on the NV, whose magnitude is estimated in 16 G.

We can nevertheless determine both ϕ and θ by generating a rotating magnetic field in the $x'y'$ plane while keeping its intensity constant. Figure S4 shows the result of such a scan with the fit indicating the orientation angles; it can be noticed that the measurement allows us to calculate also eventual residual DC fields acting on the NV center spin.

AC Superparamagnetic Nanoparticle Response

Under the effect of an external magnetic field **B** and a negligible magnetocrystalline anisotropy, the superparamagnetic nanoparticle average magnetization - in the thermal equilibrium state is governed by the Langevin equation[2]:

$$\langle m \rangle_B = mL\left(\frac{mB}{k_b T}\right) = m\left[Coth\left(\frac{mB}{k_b T}\right) - \frac{k_b T}{mB}\right] \tag{8}$$

with T being the temperature, k_b the Boltzmann constant and the particle energy in the field B being $U = -mB$. Here, m is the particle's total (or saturation) magnetic moment, calculated by adding the contributions of each crystal unit cell V_u within the volume V_p:

$$m = 32\mu_B \frac{V_p}{V_u} = 32\mu_B \frac{4\pi r^3}{3a^3} \tag{9}$$

For the spherical magnetite iron oxide nanoparticles used in our experiment, the unit cell V_u has a net magnetization of $32\mu_B$ and a lattice constant of $a = 0.839$ nm, and the single particles have an average radius of 5.5 ± 0.5 nm. The magnetic field generated by the particle's point-dipole moment has axial symmetry and can be straightforwardly calculated at the position **r** as:

$$\mathbf{B}(\mathbf{r}) = \frac{\mu_0}{4\pi}\left[-\frac{\mathbf{m}}{\mathbf{r}^3} + \frac{3(\mathbf{m}\cdot\mathbf{r})\mathbf{r}}{\mathbf{r}^5}\right] \tag{10}$$

It has to be noticed that magnetite iron oxide nanoparticles are not isotropic as according to the initial assumption, but rather uniaxial, and their energy term must take into account internal ordering effects having the form of $U = KV sin^2(\theta) - mBcos(\theta)$, with K being the magnetocrystalline anisotropy constant (in this specific case $\approx 1.4 \cdot 10^4 J/m^3$), V the particle volume, and θ the angle between the anisotropy axis and the external magnetic field. It is also fundamental to observe that anisotropy, also for

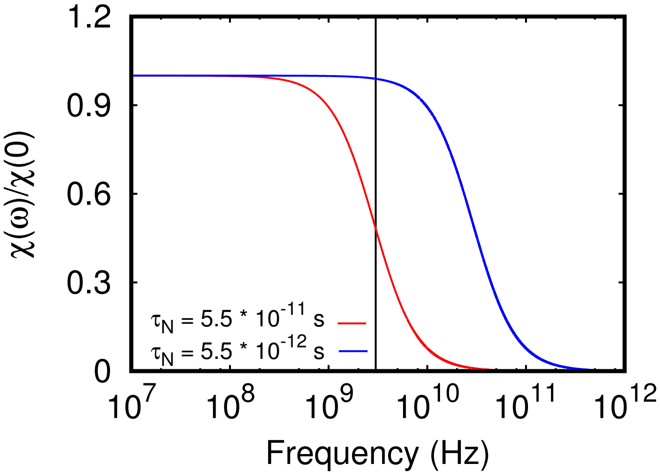

Figure S5. Real component of the first order magnetic susceptibility as a function of the field frequency, according to the Casimir-DuPre model and for $\tau_N = 5.5 \cdot 10^{-11}$ s (red line) and $\tau_N = 5.5 \cdot 10^{-12}$ s (blue line). The solid black line marks the frequency of 2.855 GHz.

$B \rightarrow 0$, arises from specific atomic/lattice configurations that would result in a net magnetization due to spin alignment within the structure (ferromagnetism). Nevertheless, when the particle volume shrinks, KV turns small or comparable to k_bT and the thermal effects become sufficiently important to induce a magnetization reversal/relaxation in the form of an exponential decay towards an equilibrium state, with a characteristic timescale described in the Neels theory[3] as:

$$\tau_N = \tau_0 e^{\frac{KV}{k_bT}} \tag{11}$$

Where τ_0 is the inverse of the attempt frequency f_0. Now, the superparamagnetic behavior appears when, for a given measurement time τ_m, the condition $\tau_N << \tau_m$ is satisfied - that is, the relaxation of the magnetization occurs on a much faster timescale than the observation process, leading to an effective null net polarization. In this case, the exact magnetic dynamics of single domains shows far more complexity and will not be treated in details here. A fundamental analysis has been performed by Brown[4] who modeled the particle magnetization through a Landau-Lifshiz-Gilbert equation with an additional stochastic term and solved the resulting Fokker-Planck equation under certain assumptions. The literature has been since then greatly expanded and several authors have discussed a manifold of different physical situations involving magnetic single domains including nonlinear responses[5,6], also in AC and DC bias fields. In our experimental conditions, with the particles diameter being in the range of 10 - 12 nm, we observe that $KV \simeq 0.73 - 1.26 \cdot 10^{-20} J$, $k_bT \simeq 4.1 \cdot 10^{-21} J$ and $mB \simeq 3.1 - 5.4 \cdot 10^{-22} J$, which implies $KV > k_bT$ and $mB < k_bT$. The inverse of the attempt frequency τ_0, which exhibits in general a dependence from temperature or external fields and their orientation respect to the easy axes[7], is assumed to be constant, since the temperature during the experiment is constant too and moreover $mB < k_bT < KV$. From the literature, we consider τ_0 to be typically in the range between 10^{-9} and $10^{-13} s$ and for our calculations we will set $\tau_0 = 5 \cdot 10^{-12} s$. In our case, we have $KV > k_bT$ and hence the Neel-Arrhenius picture should provide a sufficiently accurate description of the particle's physics. In order to have an insight in the SPION behavior in high frequency oscillating magnetic field, we adopted a less detailed approach based on the Casimir-DuPre model[8] (analog to the Debye model for electric dipoles) Here the first order AC magnetic susceptibility $\chi = \frac{\partial M}{\partial H}$ is calculated as:

$$\chi(\omega) = \chi'(\omega) + i\chi''(\omega) \tag{12a}$$

$$\chi'(\omega) = \frac{\chi(0)}{1+\omega^2\tau^2} \tag{12b}$$

$$\chi''(\omega) = \frac{\chi(0)\,\omega\tau}{1+\omega^2\tau^2} \tag{12c}$$

Where $\chi'(0)$ is the DC susceptibility, $\chi'(\omega)$ accounts for the in-phase linear response, $\chi''(\omega)$ for the out-of-phase dissipative processes, ω is the driving field frequency, and τ is the relaxation time of the magnetization. In our experiment, we have that $\tau = \tau_N$ and we can obtain the indicative plot in Figure S5. By using the NV center $m_s = 0$ to $m_s = -1$ resonant radio frequency (ω_{NV}) to manipulate both the NV electron spin and the particle magnetization, we can project its linear in-phase response on

the nitrogen-vacancy spin coherence. This means that the NV spin flip-flops will be effectively driven by the external applied AC field and the field generated by the AC nanoparticle response:

$$B_{xy}cos\left(\omega_{NV}t\right)=B_{AC,xy}cos\left(\omega_{NV}t\right)+B\left(\langle m\rangle_{B_{AC}},\mathbf{r}\right)cos\left(\omega_{NV}t\right)sin\left(\phi\right) \tag{13}$$

where $B_{AC,xy}$ is the NV-axis transversal component of the AC field, **r** the SPION-NV distance and ϕ the angle between the NV center quantization axis and B_{AC}. Knowing that $B_{AC}=\mu_0 H_{AC}$, the particles average magnetic moment can be expressed as:

$$\langle m\rangle_{B_{AC}}=\chi'\left(\omega_{NV}\right)H_{AC} \tag{14}$$

and by using the expression (8) and the equality $\chi\left(0\right)=m\left(\partial L/\partial H\right)$:

$$\langle m\rangle_{B_{AC}}=\frac{m}{1+\omega_0^2\tau_N^2}\frac{\partial L\left(\mu_0 mH/k_bT\right)}{\partial H}H_{AC} \tag{15}$$

which is equivalent to:

$$\langle m\rangle_{B_{AC}}=\frac{m}{1+\omega_0^2\tau_N^2}\frac{\partial L\left(mB/k_bT\right)}{\partial B}B_{AC} \tag{16}$$

In our experimental setting the NV center axis has an angle of ϕ = 18 ± 1 deg with respect to the z axis that defines the AC field direction - as from Figure S2 and the articles Figure 1 and 5. With a Rabi frequency of 7.33 ± 0.05 MHz, we obtain that $B_{AC}=12.0\pm0.1$ G. Concerning the superparamagnetic nanoparticle, at T = 296 K we have that $\tau_N=11\ \tau_0$ and assuming $\tau_0=5\cdot10^{-12}$ s we get a $\chi'\left(\omega_{NV}\right)$ in the range of [0.5, 1.0] as in Figure S5. Moreover, from equation (15) we can derive $\langle m\rangle\simeq1.3\times10^3\mu_b$ for our selected $B_{AC,xy}$ value. We selectively displace the SPION via AFM until it is located a distance of $<$ 100 nm from the nanodiamond. Keeping all the other experimental parameters constant, we observe a reduction of the Rabi frequency of 90 ± 70 KHz, to 7.24 ± 0.05 MHz, with the ODMR resonance frequency for the selected NV spin transition remaining in the range between [2854.5 to 2854.9] MHz. This small but measurable difference can be indeed explained by the effect of the particle average magnetization. By combining the eq. (16) with the particle characteristic physical parameters, it is possible to show that a non-zero in-phase AC magnetic field is generated by the SPION in a classical dipolar pattern. Let's set the origin of our reference frame in the center of the SPION; if the driving field is oriented along z', the magnetized particle will generate a field that, projected on the NV center xy plane, will have an opposite direction respect to the driving field, with a xy intensity at $z'=0$ nm (remembering the angles defined in the previous chapter) going from 47 mG at a distance of 50 nm to 11 mG at 80 nm, which would account for a Rabi frequency decrease of respectively 93 KHz and 22 KHz (see supplementary Figure S6). By choosing any other plane between $z'=0$ and $z'=45$ nm (remembering the nanodiamond having a radius of 20 to 25 nm and the SPION of 5.5 nm) we obtain the graph as in the main article Figure 1, where the effective counter-active field is in the order of magnitude of several 10^{-2} G. Finally, we may consider that, despite not having a precise localization of the NV center, our simulations and results agree with its localization within the spherical particle representing the nanodiamond and located between 50 and 80 nm from the SPION.

Relaxometry of a Superparamagnetic Nanoparticle

An NV center T_1 time measurement (also denoted as relaxometry measurement) can be used to detect a magnetic field noise acting on the spin population of the defect and having a frequency component comparable to the $m_s=0\rightarrow m_s=\pm1$ transition energies. The general treatment of this problem, that is an individual spin subject to a random fluctuating magnetic field, can be found in several sources[9,10]. In our case we start by taking the NV center S = 1 quantum system and defining the Z axis as the quantization axis determined by the large zero-field splitting. We prepare, by laser polarization, the center's spin in the $m_s=0$ state. Now, the dynamics of the center spin may be modeled with a system of rate equations - excluding the intersystem crossing to the dark state:

$$\begin{pmatrix}n'_0\\n'_{+1}\\n'_{-1}\end{pmatrix}=\begin{pmatrix}-2k&k&k\\k&-k&0\\k&0&-k\end{pmatrix}\begin{pmatrix}n_0\\n_{+1}\\n_{-1}\end{pmatrix} \tag{17}$$

where n_0, n_{+1} and n_{-1} are the populations of the $m_s=0,+1,-1$ spin levels obeying the normalization relation $n_0+n_{+1}+n_{-1}=1$. By solving the differential equation system it can be found that n_0 relaxes over time with an exponential decay towards the equilibrium condition:

$$n_0=-2Ce^{-3kt}+\frac{1}{3} \tag{18}$$

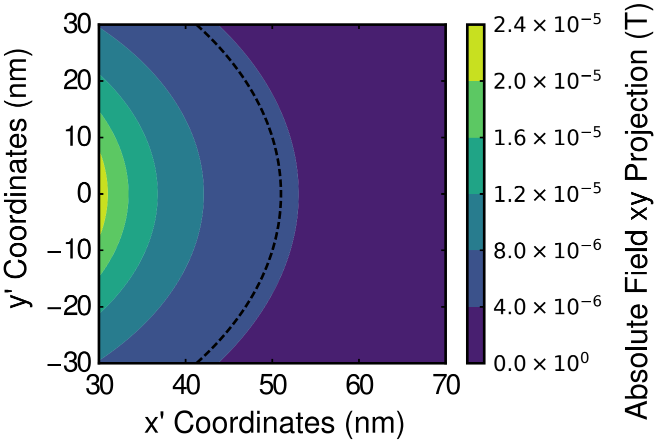

Figure S6. Visualization of the field generated by the SPION that is effectively driving the NV center electron spin as a function of the distance from the SPION center (set in the origin of the reference system). The x'y' system of coordinates is given by the Helmholtz coils (x'y' is equiplanar to the waveguide surface as well) and z' is parallel to the driving microwave field. The plot is calculated assuming z' = 0, that is considering the NV center and the SPION center to lie on the same plane parallel to the surface. In this case, the SPION AC response is anti-parallel with respect to the external driving AC field. The dashed line shows the coordinates where the SPION AC response has an effective strength on the NV center xy plane of respectively 45 mG.

with $3k = 1/T_1$ and C being a constant dependent on the boundary conditions. We continue by observing that in our experiment, after the initial polarization, the NV center spin interacts with the SPION magnetization vector whose dynamics is described by a stochastic process with a zero first-order momentum (*i.e.* zero-average), a non-zero second-order momentum (variance) and a exponentially decaying autocorrelation function. In this case, we can relate the spin population decay constant with the variance of the SPION transversal magnetization with:[9,10]

$$\frac{1}{T_1} = 3k = 3\gamma_{NV}^2 \langle B_{xy}^2 \rangle A\left(\omega_{NV}, \tau_N\right) \tag{19}$$

where:

$$A\left(\omega, \tau_N\right) = \frac{\tau_N}{1 + \omega_{NV}^2 \tau_N^2} \tag{20}$$

Finally, by combining the above equations with the intrinsic relaxation rate of the NV center, we derive:

$$\frac{1}{T_1} = \frac{1}{T_{1,NV}} + 3\gamma_{NV}^2 \langle B_{xy}^2 \rangle \frac{\tau_N}{1 + \omega_{NV}^2 \tau_N^2} \tag{21}$$

Now, in order to derive from the variance measurement the total particle magnetic moment and its distance from the defect center, we proceed by calculating the statistical second order momentum of the magnetic field components generated by the particle dipole at a position **r** as from the equation (10), and integrate with a weighting term over a spherical surface to account for the random fluctuations. Considering an angle κ between the NV z (quantization) axis and the magnetic particle-NV direction vector, and given $\langle B_{xy}^2 \rangle = \langle B_x^2 \rangle + \langle B_y^2 \rangle$ we get:

$$\langle B_{xy}^2 \rangle = \frac{m^2 \mu_0^2}{48\pi^2} \left[\frac{5 - 3cos^2\left(\kappa\right)}{r^6} \right] \tag{22a}$$

$$\langle B_z^2 \rangle = \frac{m^2 \mu_0^2}{48\pi^2} \left[\frac{1 + 3cos^2\left(\kappa\right)}{r^6} \right] \tag{22b}$$

Our experimentally observed $\langle B_{xy}^2 \rangle$ is 2.9 ± 0.5 G^2, and given an angle $\kappa \simeq 72 \pm 1$ deg as from the orientation measurements, we get a magnetic moment of 38000 ± 6000 μ_b for an in-plane (x'y') separation of 64 nm (here, the moment uncertainty arises from the T_1 measurement errors and the NV axis angle estimation error). However, it can be noticed that given the different sizes of the nanodiamond and the SPION, we may have different combinations of vertical (z') and in-plane (x'y')

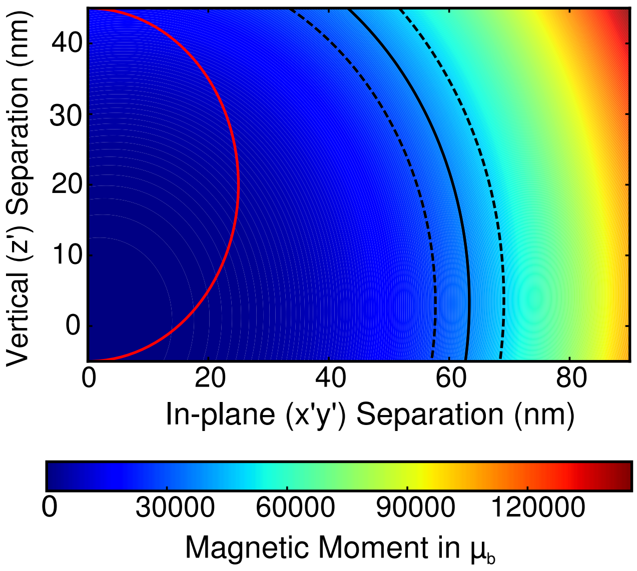

Figure S7. Plot of the expected magnetic moment for the SPION as a function of the (x'y') and z' projected distance with respect to the NV center in the diamond. The red circumference shows the nanodiamond (only for comparative purposes). The black solid line represents the coordinates that correspond to a 38000 μ_b SPION that is compatible with our particle diameter of 11 nm, while the dashed lines show the coordinate uncertainty area given an uncertainty on the particle size of $\pm$ 1 nm as from the AFM measurements.

separations that correspond to a particle of a defined size (or magnetic moment) interacting with the NV. In other words, the change observed in the T_1 time could come from a 38000 μ_b particle located at $\kappa = 72°$ and $r_{x'y'} = 64$ nm or from the same particle positioned at $\kappa = 95°$ and $r_{x'y'} = 59$ nm or anywhere else on the curve $f(r,\kappa)$ that satisfies the equations in (22). In Figure S7 we show the estimated magnetic moment of the iron oxide nanoparticle as a function of the position with respect to the NV center, where the solutions for a particle of radius R = 5.5 $\pm$ 0.5 nm are highlighted.

Hahn-echo Fit

The Hahn-echo decays have been fitted using the formula:

$$f(t) = A + B\exp\left[-\left(\frac{t}{T_2}\right)^2\right] \quad (23)$$

where A is the offset parameter, B the contrast, T_2 the transverse decay time. Now, the overall relaxation rate T_2 can be considered as the sum of the "bare" NV center relaxation rate and the SPION contribution:

$$\frac{1}{T_2} = \frac{1}{T_{2,NV}} + \frac{1}{T_{2,SPION}} \quad (24)$$

where $1/T_{2,SPION}$ is equal to (see equations 19-21 and the main text reference 46):

$$\frac{1}{T_{2,SPION}} = \frac{1}{2T_1} + \gamma_{nv}^2 \langle B_z^2 \rangle \tau_N \quad (25)$$

with $\langle B_z^2 \rangle$ being the variance of the B field component parallel to the NV center axis. In order to solve this equation in our practical case, we need to use the T_1 obtained from the relaxometry measurement, and calculate $\langle B_z^2 \rangle$ from the estimated NV-SPION distance and particles magnetic moment. By doing that, we may observe that the expected $1/T_{2,SPION}$ contribution to $1/T_2$ is at least one order of magnitude lower with respect to the bare NV center contribution, which would translate in a modest (circa 140 ns) reduction of the overall T_2 time.

References

1. Doherty, M.W., Michl, J., Dolde, F., Jakobi, I., Neumann, P., Manson, N. B. & Wrachtrup, J. Measuring the defect structure orientation of a single NVcentre in diamond. *New Journ. Phys.* **16**, 063067 (2014).
2. Coffey, W., Kalmykov, Y. P., Waldron, J. T. *The Langevin equation: with applications to stochastic problems in physics* (World Scientific, 2005).
3. Neel, L. Théorie du trainage magnétique des ferromagnétiques aux grains fins avec applications aux terres Cuites. *Annu. Geophys.* **5**, 99-136 (1949).
4. Brown, W. F. Thermal fluctuations of a single-domain particle. *Phys. Rev.* **130**, 1677-1686 (1963).
5. Jönsson, P., Jonsson, T., Garcia-Palacios, J. L. & Svedlindh, P. Nonlinear dynamic susceptibilities of interacting and noninteracting magnetic nanoparticles. *Journ. Magn. Magn. Mat.* **222**, 219-226 (2000).
6. El Mrabti, H., Titov, S. V., Déjardin, P.-M. & Kalmykov, Y. P. Nonlinear stationary AC response of the magnetization of uniaxial superparamagnetic nanoparticles. *Journ. Appl. Phys.* **110**, 023901 (2011).
7. Wang, X. & Bertram, H. N. Field and temperature-dependent attempt frequency for dynamic-thermal reversal of interacting magnetic grains. *J. Appl. Phys.* **92**, 8 (2002).
8. Casimir, H. B. G. & du Pré, F. K. Note on the thermodynamic interpretation of paramagnetic relaxation phenomena. *Physica* **5**, 507-511 (1938).
9. Levitt, M. *Spin dynamics*, 543-552 (John Wiley & Sons, 2008).
10. Slichter, C. P. *Principles of magnetic resonance*, 197-198, 209-212 (Springer, 1990).
